# Supplementary material for: Developmental series of gene expression clarifies maternal mRNA provisioning and maternal-to-zygotic transition in a reef-building coral
Source: BMC Genomics. 2021 Nov 11;22:815. doi: 10.1186/s12864-021-08114-y (PMC8588723; doi:10.1186/s12864-021-08114-y)
Supplement: Supplementary file 2 — Additional file 2 [file 12864_2021_8114_MOESM2_ESM.docx]

**Developmental series of gene expression clarifies maternal mRNA provisioning and maternal-to-zygotic transition in a reef-building coral**

Chille E, Strand E, Neder M, Schmidt V, Sherman M, Mass T, Putnam HM

##

## Table of Contents

[Supplementary Materials and Methods](#_heading=h.1fob9te) 1

[Experimental Conditions](#_heading=h.2et92p0) 1

[Functional annotation methodology and results](#_heading=h.tyjcwt) 2

[Local Alignment Search for Developmental Biomarkers](#_heading=h.1t3h5sf) 2

[Supplementary Figures](#_heading=h.4d34og8) 3

[References](#_heading=h.17dp8vu) 3

## Supplementary Materials and Methods

### Experimental Conditions

Immediately after collection, 300 uL of egg and sperm bundles were snap frozen and stored at -80 °C while the rest of the bundles were placed in conical chambers to break apart and hydrate for 10 minutes. 300 uL of eggs were separated from the sperm and rinsed 3 times with 0.2 uM filtered seawater then snap-frozen and stored at -80 °C. Fertilization and early development (0 h - ~16 hfp) took place in 3 replicated 1.6L flow through conical chambers and further development (~ 16 hpf - 9 d) took place in small (6 x 6 cm) flow through bins within larger 74L flow through tanks, both under ambient temperature ~26.8°C. Water flow to the cultures was controlled with ½ GPH Pressure Compensating Drippers and held at a maximum potential flow rate of 7.57 liters per hour. For a 1.6 L conical, the turnover rate was once every ~50 minutes and for a 74L tank, the turnover rate was once every ~ 23 hours. The average light intensity of the experimental setup was measured using a handheld PAR sensor (Underwater Quantum Flux Apogee instruments - Model MQ-510; accuracy = ±4%) and was 115.8 ± 14.7 µmol m^-2^ s^-1^, n=6 measurements. Temperature, salinity, and pH were measured three times daily using a handheld digital thermometer (Fisherbrand Traceable Platinum Ultra-Accurate Digital Thermometer, accuracy = ±0.05 °C, resolution = 0.001°) and a portable multiparameter meter (Thermo Scientific Orion Star A-series A325; accuracy = ±0.2 mV, 0.5% of PSU reading, resolution = 0.1 mV, 0.01 PSU) with pH and conductivity probes (Mettler Toledo InLab Expert Pro pH probe #51343101; Orion DuraProbe 4-Electrode Conductivity Cell Model 013010MD), respectively. Temperature and pH probes were calibrated using Tris (Dickson Laboratory Tris Batch 27, Bottles 70, 75, 167, 245, and 277) standard calibrations. Throughout the experiment, the temperature, salinity, and pH of tanks and conicals (mean±SEM) was 26.99±0.075°C, 34.24±0.017 psu, and -52.84±0.348 mV, respectively.

### Functional annotation methodology and results

Comprehensive gene ontology (GO) annotation of the reference genome was undertaken for subsequent functional enrichment analysis using InterProScan (v.5.46-81.0), Blast2GO (v5.2), and UniProt [[1–3]](https://paperpile.com/c/F91Y1v/l5e1+rN2N+AwAl). First, homologous protein sequences were identified using the DIAMOND (v2.0.0) blastx program in “more sensitive” mode to map predicted cDNA sequences against the NCBI non-redundant (nr) protein database (downloaded on August 6, 2020) using an e-value cut-off of 1e-05 and a block size of 20 [[4]](https://paperpile.com/c/F91Y1v/hwEr). Concurrently, InterProScan’s *iprlookup* function was run to map GO terms from the InterPro database (accessed on August 24, 2020) to the *M. capitata* predicted protein sequences [[2, 5]](https://paperpile.com/c/F91Y1v/rN2N+T7K6). Next, the XML output files from DIAMOND and InterProScan were both loaded into Blast2GO for compilation and further mapping using the obo database (updated August 11, 2020) [[1]](https://paperpile.com/c/F91Y1v/l5e1). Additional GO terms were extracted from the UniProtKB database by searching for protein identifiers obtained via DIAMOND using UniProt’s “Retrieve/ID mapping tool” [[3]](https://paperpile.com/c/F91Y1v/AwAl). Finally, mapping results from Blast2GO and UniProt were compiled in RStudio (v1.3.959) [[6]](https://paperpile.com/c/F91Y1v/6DaH), using R (v-4.0.2) [[7]](https://paperpile.com/c/F91Y1v/1MqR).

Sequence alignment using DIAMOND resulted in 55,217 significant pairwise alignments for the total 63,227 sequences queried, with a median e-value of 3.1e-72 and a median bitscore of 282.3. Blast2GO mapped 4,205 sequences to one or more GO terms. Additionally, a UniProt search of the protein identifiers obtained through DIAMOND matched 2,351 sequences with one or more GO terms (7,408 total terms). Finally, annotation with InterProScan matched 49,338 out of the 63,227 query sequences to entries in the InterProScan databases, 20,603 of which were associated with one or more GO terms (47,726 total terms). In total, 3,264 unique GO terms were mapped to 23,107 genes, with a total of 55,974 annotations to the *M. capitata* transcriptome (Table S8).

### Local Alignment Search for Developmental Biomarkers

The temporal expression patterns of enzymes with putative roles in the MZT (Cyclin-B, Smaug, Kaiso, Sox2, Wnt8, and TBXT) and transcriptional regulation (DNMT1, DNMT3A, TET1, MBD2, MBD3, UHRF1, and BRG1) were assessed to provide context to the global gene expression patterns observed during *M. capitata* development. To accomplish this, anthozoan (as available) and model organism protein sequences for selected enzymes were identified from the NCBI protein database for a local alignment query. Sequence length, organism, quality, and date modified were considered in cases for which there were multiple available sequences. Blastx [[8]](https://paperpile.com/c/F91Y1v/tJ3Z) was used to identify the enzymes of interest in the *M. capitata* reference transcriptome (e-value < 10−5, max_target_seqs=100) using a Blast database created from the predicted cDNA sequences [[5]](https://paperpile.com/c/F91Y1v/T7K6). The hits table was filtered for duplicates in R. This table was used to subset the vst-normalized gene counts matrix, leaving only hits to the enzymes of interest. The top hits for each enzyme (Table S1) generally exhibited much higher bitscores and lower e-values compared to subsequent hits. However, in some cases, the blast results and expression profiles for the top 2-3 hits were nearly identical, even though the genes were located on different scaffolds in the genome. This may be attributed to the high gene duplication reported for the reference *M. capitata* genome [[5]](https://paperpile.com/c/F91Y1v/T7K6) used here.

## Supplementary Figures

**Figure S1.** **Boxplot and overlaid points of mean eigengene expression value of each of the replicate samples** per time point (n=3, except mid-gastrula and late-gastrula where n=2) for each WGCNA module cluster 1-9.


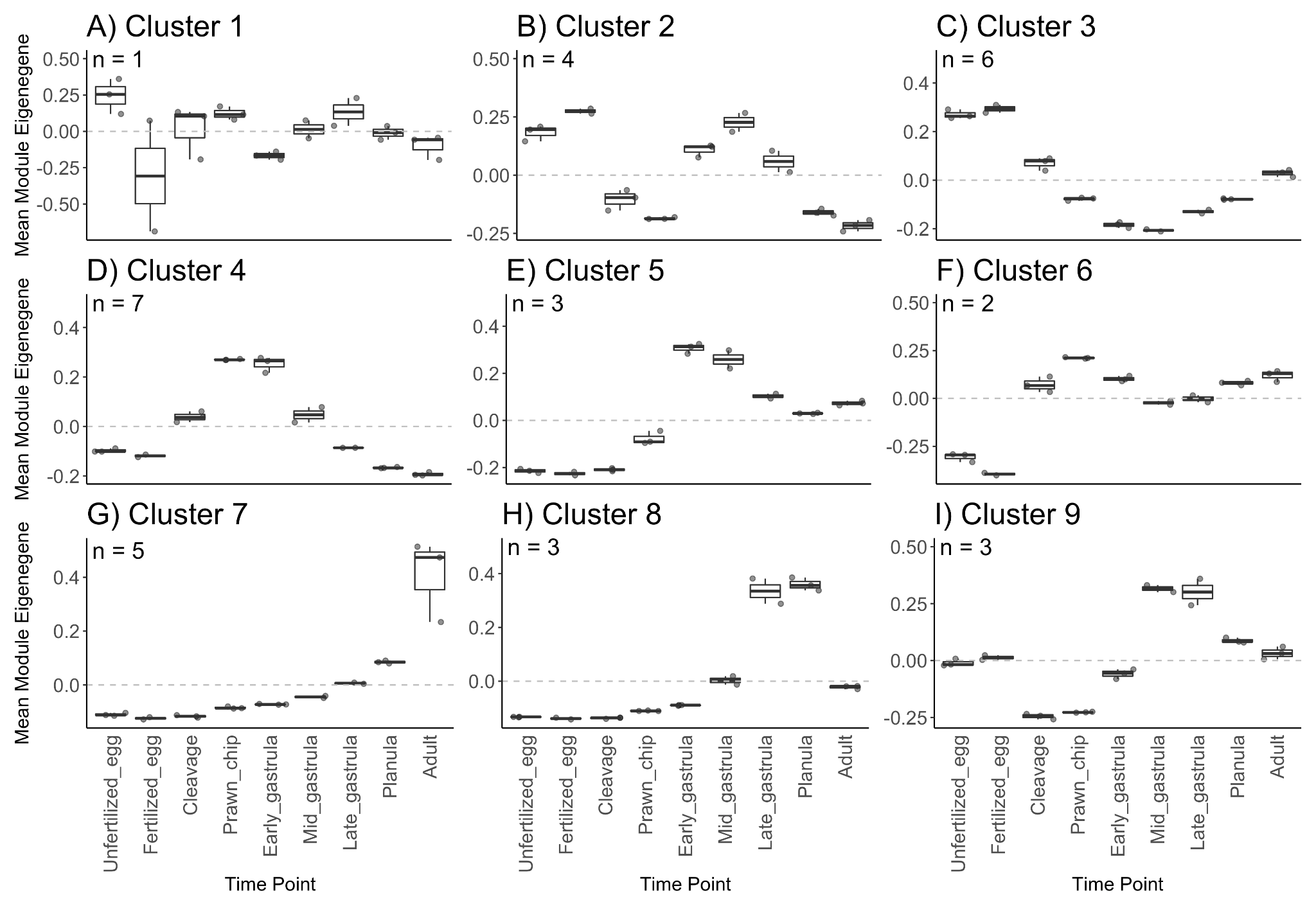


## References

[1. Götz S, García-Gómez JM, Terol J, Williams TD, Nagaraj SH, Nueda MJ, et al. High-throughput functional annotation and data mining with the Blast2GO suite. Nucleic Acids Res. 2008;36:3420–35.](http://paperpile.com/b/F91Y1v/l5e1)

[2. Jones P, Binns D, Chang H-Y, Fraser M, Li W, McAnulla C, et al. InterProScan 5: genome-scale protein function classification. Bioinformatics. 2014;30:1236–40.](http://paperpile.com/b/F91Y1v/rN2N)

[3. UniProt Consortium. UniProt: a worldwide hub of protein knowledge. Nucleic Acids Res. 2019;47:D506–15.](http://paperpile.com/b/F91Y1v/AwAl)

[4. Buchfink B, Xie C, Huson DH. Fast and sensitive protein alignment using DIAMOND. Nat Methods. 2015;12:59–60.](http://paperpile.com/b/F91Y1v/hwEr)

[5. Shumaker A, Putnam HM, Qiu H, Price DC, Zelzion E, Harel A, et al. Genome analysis of the rice coral Montipora capitata. Sci Rep. 2019;9:2571.](http://paperpile.com/b/F91Y1v/T7K6)

[6. RStudio Team. RStudio: Integrated Development for R. Boston, MA: RStudio, PBC; 2020.](http://paperpile.com/b/F91Y1v/6DaH) <http://www.rstudio.com/>[.](http://paperpile.com/b/F91Y1v/6DaH)

[7. R Core Team. R: A language and environment for statistical computing. 2013.](http://paperpile.com/b/F91Y1v/1MqR) <https://cran.microsoft.com/snapshot/2014-09-08/web/packages/dplR/vignettes/xdate-dplR.pdf>[.](http://paperpile.com/b/F91Y1v/1MqR)

[8. Altschul SF, Gish W, Miller W, Myers EW, Lipman DJ. Basic local alignment search tool. J Mol Biol. 1990;215:403–10.](http://paperpile.com/b/F91Y1v/tJ3Z)
